# Advances in the Use of Regulatory T-Cells for the Prevention and Therapy of Graft-vs.-Host Disease

**DOI:** 10.3390/biomedicines5020023

**Published:** 2017-05-16

**Authors:** Reshma Ramlal, Gerhard C. Hildebrandt

**Affiliations:** Division of Hematology and Bone Marrow Transplantation, University of Kentucky, Markey Cancer Center 800 Rose Street, Lexington, KY 40536, USA; gerhard.hildebrandt@uky.edu

**Keywords:** Tregs, graft versus host disease (GvHD), adoptive cellular therapy, CD4+CD25+FoxP3+ T cells

## Abstract

Regulatory T (Tregs) cells play a crucial role in immunoregulation and promotion of immunological tolerance. Adoptive transfer of these cells has therefore been of interest in the field of bone marrow and solid organ transplantation, autoimmune diseases and allergy medicine. In bone marrow transplantation, Tregs play a pivotal role in the prevention of graft-verus-host disease (GvHD). This has generated interest in using adoptive Treg cellular therapy in the prevention and treatment of GvHD. There have been several barriers to the feasibility of Treg cellular therapy in the setting of hematopoietic stem cell transplantation (HSCT) which include low Treg concentration in peripheral blood, requiring expansion of the Treg population; instability of the expanded product with loss of FoxP3 expression; and issues related to the purity of the expanded product. Despite these challenges, investigators have been able to successfully expand these cells both in vivo and in vitro and have demonstrated that they can be safely infused in humans for the prevention and treatment of GvHD with no increase in relapse risk or infections risk.

## 1. Introduction

Immunosuppressive therapy with calcineurin inhibitors with low dose methotrexate or with mycophenolate mofetil has formed the mainstay of graft versus host disease (GvHD) prevention in allogeneic hematopoietic stem cell transplantation (HSCT) recipients for decades. Moreover, corticosteroids remain the major pharmacotherapeutic treatment of GvHD [1]. Nevertheless, GvHD still develops in 40–60% of recipients and remains a major cause of non-relapse mortality [2]. As a result, there is a need for the development of more effective strategies to mitigate GvHD.

One approach that is currently being investigated is the use of naïve CD4+CD25+FoxP3+ regulatory T cells (nTregs) in the prevention and treatment of GvHD. These cells are thymic derived and account for 5–10% of the peripheral CD4+/CD8− cells in humans [3]. They demonstrate suppressor activity and play a pivotal role in self-tolerance maintenance and prevention of auto-immune diseases [4]. Induced Tregs are generated in peripheral lymphoid organs following adequate antigenic stimulation in the presence of cognate antigen and specialized immunoregulatory cytokines such as transforming growth factor beta, interleukin-4, interleukin-10.

## 2. The Role of nTregs in the Graft Versus Host Disease (GvHD) Biology

GvHD is characterized by an imbalance of the effector and regulator arms in the immune system resulting in the over-production of inflammatory cytokines [5]. The conditioning regimen prior to HSCT, usually consisting of chemotherapy with or without total body irradiation, produces host organ injury which leads to the release of pro-inflammatory cytokines. These cytokines stimulate host antigen presenting cells, which present host peptides to donor T cells in the graft. These peptides are recognized as foreign and result in T cell activation early after transplant. Donor naïve T cells differentiate into cytokine producing effector T cells with predominantly a Th1 or Th17 profile for CD4 T cells and with a Tc1 (Type 1 cytotoxic T-cell) profile for CD8+ effector cells [6]. In a positive feedback loop mediated by cytokine storm and tissue injury-mediated upregulation of chemokines, more effector cells such as cytotoxic T cells, NK cells and macrophages are recruited, leading to direct organ damage that typically affects the gut, liver and skin [7]. The pro-inflammatory milieu results in a reduced number of Tregs as the naïve T cells preferentially differentiate into effector T cells in the presence of interleukin-6 (IL-6) and transforming growth factor -β (TGF-β) [8,9]. Furthermore, IL-6 is able to inhibit the suppressor function of nTregs through the activation of Toll-like receptors [10].

nTregs mediate their suppressive function through contact-dependent and contact-independent mechanisms. Contact-dependent mechanisms involve the expression of inhibitory molecules such as cytotoxic T-lymphocyte-associated protein 4 (CTLA-4), lymphocyte-activation gene 3 (*LAG-3*) and Neuropilin-1 which inhibit co-stimulatory markers resulting in decreased proliferation of T cells. Contact-independent mechanisms of immune regulation involve the secretion of anti-inflammatory cytokines such as interleukin 10 (IL-10) and TGF-β and interleukin-35 (IL-35) [11].

Taylor et al. [12], using murine models, demonstrated the role of nTreg in the biology of GvHD. They demonstrated that CD4+CD25+ depletion from a donor T cell inoculum or in vivo CD25 depletion of the recipient resulted in accelerated GvHD mortality in vivo. Furthermore, they showed that ex vivo activated and expanded CD4+CD25+T cells resulted in significant improvement in GvHD.

The role of nTreg in mitigating the risk of GvHD has also been demonstrated in humans. Using HLA-matched related donor (MRD) transplants, Rezvani et al. [13] demonstrated that a high CD4+CD25+FoxP3+ T cell count in the donor graft was associated with a reduced risk of GvHD. They also found that low nTreg cells early after allogeneic HSCT were associated with an increased risk of GvHD. It has been shown that patients with active chronic GvHD have reduced frequency of Tregs compared to healthy volunteers, again demonstrating an association with Treg deficiency and GvHD [8].

Apart from mediating GvHD, alloreactive cytotoxic T cells are also responsible for the graft-versus-tumor effect (GvT). Suppressing the activity of these cells can potentially abrogate the GvT effect. However, it has been demonstrated in vitro that nTregs did not inhibit the ability of the allostimulated CD8+ T cells to kill tumor cells. This finding was confirmed in murine models by Edinger and colleagues [14]. The ability of nTcells to mitigate the effects of GvHD while preserving the GvT activity makes it an attractive option for adoptive cellular therapy approaches.

## 3. nTreg Expansion

Some of the challenges that have plagued the development of adoptive nTreg cellular therapy have been the low frequency of these cells in the peripheral blood as well as their relative anergy. It has been demonstrated that ratios of 1:1 and 1:3 of T effector cells:T reg cells are needed in order for there to be a marked reduction in murine GvHD [15]. nTreg cells need to undergo expansion and reach sufficient quantity to have an impact on GvHD outcomes. Expansion of the nTreg cells can be done through in vivo stimulation of nTreg proliferation or through ex vivo expansion.

### 3.1. In Vivo Expansion for GVHD Prophylaxis

nTregs express high levels of the interleukin (IL)-2 receptor alpha CD25. Based on this, Kennedy-Nasser and colleagues hypothesized that nTreg can be selectively expanded in vivo in response to low doses of IL-2 that are insufficient to stimulate T effector cells [16]. To demonstrate this, they prospectively treated sixteen pediatric patients with ultralow doses (ULD) of IL2 (100,000–200,000 IU/m^2^) three times per week starting between days 7 and 30 post-transplantation and continuing for up to twelve weeks. No grade 3/4 toxicities were associated with ULD IL-2. An increase in the frequency of CD4+CD25+FoxP3+ Tregs from baseline was seen in all patients from a mean of 4.8% pre-IL-2 to 11.1% following 6 weeks of IL-2 therapy. These regulatory T cells suppressed alloreactive responses in vitro, indicating functionality. The patients in this single arm phase II study were compared to a contemporaneous group of 33 recipients treated with the same conditioning regimen and GvHD prophylaxis as the IL-2 study patients, except for the exclusion of IL-2. No. IL-2 treated patients developed grade 2–4 acute GvHD compared to 12% in the control arm. There was also a statistically significant reduction in the incidence of viral infections in the matched related donor IL-2 recipients (15%) versus 63% in the comparison group (*p* = 0.022).

In vivo Treg expansion is an attractive option for obtaining a nTreg cell dose sufficient to mitigate the effects of GvHD since it is relatively inexpensive and does not require extensive cell sorting and cell culture compared to ex vivo T cell expansion.

Other potential targets for in vivo expansion of nTreg include the TNF Receptor Superfamily Member 25 (TNFRSF25). Wolf et al. demonstrate massive but transient Treg expansion in donor mice (>50% of CD4+ compartment) when this receptor was stimulated with the TL1A-Ig fusion protein together with IL-2. Tregs increased in multiple compartments including blood, lymph nodes, spleen and colon. However, no Treg expansion was seen in the bone marrow which is a critical site for the GvT response. In these murine models, when transplants were performed across HLA-identical minor antigen mismatched donor–recipient pairs, there was a dramatic reduction of GvHD with sparing of anti-tumor response [17].

### 3.2. In Vivo Expansion for Treatment of GvHD

Low dose IL-2 has also been demonstrated to be efficacious in the treatment of steroid refractory chronic GvHD (cGvHD). Koreth and colleagues treated 35 patients with steroid refractory cGvHD with daily IL-2 at a dose of 1 × 10^6^ IU/m^2^ for 12 weeks [18]. There was a 61% (20/33 patients) response rate at multiple cGvHD sites by National Institutes of Health (NIH) criteria. No patients however, achieved a complete response. In this study, there was a five-fold increase in the Treg levels when compared to pre-treatment levels without significant change in the conventional T cells (Tcons) or CD8 T cells. The clinical responders who were eligible to receive IL-2 therapy indefinitely showed durable clinical and Treg immune responses.

Extracorporeal photopheresis is one of the therapeutic modalities for GvHD, whose effects were thought to be mediated through the induction of regulatory T cells [19,20]. However, recent data suggests that this mechanism of action may not be applicable in all scenarios [21] and that other mechanisms, such as the infusion of apoptotic cells, contribute as well [22,23].

### 3.3. In Vitro Expansion for GVHD Prophylaxis

Umbilical cord blood (UCB) T cells contain a greater percentage of the CD4+CD25+ subset which are largely naïve and less likely to become effector T cells upon stimulation compared to adult T cells [24]. As such, UCB represents an attractive source of Tregs for in vitro expansion.

Brunstein et al. performed the first in human clinical trial using cord blood expanded Treg cells in adults undergoing double cord blood transplantation [25]. In their first clinical trial, Tregs isolated from a partial HLA-matched third cord blood unit underwent anti-CD3/anti-CD28 monoclonal antibody beads stimulated expansion methodology [25]. This resulted in a median 211-fold expansion with 17 of the 23 patients (74%) receiving the Treg infusion at the targeted dose of 30 × 10^5^/kg. All of the Treg product was suppressive in vitro with a median suppression of 86% at the end of culture. All 23 patients in the study underwent conditioning with cyclophosphamide 50 mg/m^2^, fludarabine 40 mg/m^2^ (days −6 to −2) and 200 cGy TBI in a single fraction on day −1. Treg cells were infused on day +1 and on day +15 in 15 of the planned 18 patients who were to receive two doses. GvHD prophylaxis consisted of mycophenolate mofetil and cyclosporine in the first 17 patients and sirolimus and mycophenolate mofetil in six patients following a protocol amendment. To assess the impact of the Treg infusion on the risk of graft failure, GvHD and non-relapse mortality, they compared the outcomes of 108 patients with hematological malignancies treated in an identical fashion.

In this study, no infusional toxicities were observed. The UCB Tregs could be detected for 14 days with the greatest portion of Treg cells present on day +2. A statistically significant reduction in grade II–IV acute GvHD was observed in the Treg group compared to the 108 identically treated historical controls (43% vs. 61%, *p* = 0.05). There were no differences observed in relapse risk, infection risk and early mortality compared to the historical control. Two of fourteen (14%) patients at risk developed chronic GvHD with no patients in the Treg dose ≥ 30 × 10^5^ /kg which is favorable compared to an incidence of chronic GvHD of 26% in the historical controls.

One issue related to the expansion of Treg cells, as described above by Brunstein et al., is related to the purity of the product. Treg isolation using immunomagnetic beads is contaminated by effector T cells [26]. Several groups are exploring the use of Treg expansion protocols which include using the drug rapamycin to reduce the undesirable expansion of effector T cells and to increase the stability of the expanded Treg product [27]. Further strategies to improve Treg purity and cell dose are through the generation of allo-antigen specific Tregs. Brunstein and colleagues [28,29] were able to generate UCB Treg doses as high as 100 × 10^6^ /kg by modifying the ex vivo expansion procedure to include a K562 antigen presenting cells expressing CD64 and CD86 instead of immunogenic CD3 and CD28 beads. A total of 11 patients were treated in this trial. No drug limited toxicities were observed in doses up to 100 × 10^6^/kg. The greater infused cell dose was associated with high peak Treg levels but did not result in persistence of the cells beyond 14 days. The transplant related outcomes of these 11 patients were compared to 22 identically treated historical controls. Here, they demonstrated a significant reduction in acute GvHD with cumulative incidence of grade II–IV aGvHD of 9% in Treg-treated patients vs. 45% in control (*p* = 0.05). None of the Treg recipients developed chronic GvHD vs. 14% in the control group. The risk of viral reactivations, cumulative risk of relapse and non-relapse mortality were similar in the Treg-treated group and control group. There appeared to be a trend of improved 1-year survival in the Treg-treated group versus control (81% versus 61%), however this was not statistically significant (*p* = 0.30).

The use of adoptive Treg cell therapy has also been demonstrated to be efficacious in the prevention of GvHD in the setting of the HLA-haploidentical HSCT without any increase in relapse risk [30,31]. Di Ianni et al. treated 28 patients with high risk hematological malignancies undergoing a HLA-haploidentical HSCT with freshly isolated donor-derived Treg infused on day −4 of the treatment protocol [30]. CD34+ cells were infused on day 0 along with the infusion of conventional T cells. No patients in this study received post-transplantation immunosuppressants. At a median follow-up of 11.2 months, no patients developed cGvHD and 2/26 evaluable patients developed ≥ grade 2 aGVHD. Furthermore, the nTregs infused did not inhibit the expansion of coinfused Tcons, allowing for rapid and sustained T-cell subpopulation reconstitution with a reduced incidence of cytomegalovirus (CMV) reactivations. The authors presented an update of these results in which a total of 52 patients with high risk acute leukemias were treated [31,32]. At a median follow up of 4-years (range, 7–58 months), the treatment-related mortality rate (TRM) was 40%, and disease-free survival rate (DFS) was 58%. Of the evaluable patients, 12% (*n* = 6 of 50) developed ≥ grade II aGvHD with only 2% (*n* = 1 of 52) developing cGvHD even though 1.1 × 10^6^/kg Tcons were infused. They also found that compared with T-cell depleted Haplo HSCT, CD4+ and CD8+, specific for opportunistic pathogens such as Aspergillus fumigatus, Candida albicans, CMV, herpes simplex virus, adenovirus toxoplasmosis, and varicella zoster virus, emerged at a significantly earlier time point. Also of significance in this study was the low rate of relapse with only 5% (*n* = 2 of 41) of evaluable patients relapsing. These studies demonstrated that ex vivo expanded T cells can be safely infused in humans, resulting in reduction in GvHD. Furthermore, Treg permitted co-transplantation of enough Tcons to eradicate minimal residual disease, resulting in reduction in post-transplantation relapse from the usual 30–35% seen in high risk leukemia without an increase in the incidence of aGvHD, while simultaneously resulting in improved immunologic reconstitution. These results are encouraging and need to be confirmed in larger prospective studies.

## 4. Future Directions

Adoptive nTreg cellular therapy represents a promising strategy for the prevention and management of GvHD. However, generation of sufficient quantities of Treg cells using ex vivo expansion techniques requires specialized expertise as well as expensive cell sorting and culturing methods which may limit its general applicability to transplant centers. Furthermore, improvements in in vitro expanded Treg cellular stability and persistence following infusions are necessary. Currently, these cells persistent on average 14 days following infusion [28]. Novel strategies combining Treg infusions with the immunosuppressive agent rapamycin or, more recently, low dose IL-2 are being investigated to prolong Treg survival and increase Treg stability in vivo [26]. There is also a need for further studies looking into the generation of “off the shelf” Treg cellular products.

In vivo nTreg expansion has the advantage of being more cost effective and easier to implement, thus favoring more widespread clinical applicability over in vitro Treg expansion. Further research geared at exploring therapies to stimulate in vivo nTreg expansion includes the use of agents such as sirolimus and retinoic acid [33] as well as novel agents such as TNF superfamily receptor TNFRSF25 [16] and liposomal synthetic derivative of α-galactosylceramide (RGI-2001) [34]. Zinc supplementation may also represent a novel, non-toxic and easily applicable means of inducing Tregs and requires further study in animal and human models [35].

In summary, regulatory T cells present a promising cell therapy product. Pre-clinical and preliminary clinical data suggest their efficacy in acute and chronic GvHD, yet clinical feasibility and applicability in routine care of GvHD patients still need to be defined.
